# miR-222 Suppresses Immature Porcine Sertoli Cell Growth by Targeting the *GRB10* Gene Through Inactivating the PI3K/AKT Signaling Pathway

**DOI:** 10.3389/fgene.2020.581593

**Published:** 2020-10-29

**Authors:** Hui Luo, Fuzhi Peng, Bo Weng, Xiangwei Tang, Yao Chen, Anqi Yang, Bin Chen, Maoliang Ran

**Affiliations:** College of Animal Science and Technology, Hunan Provincial Key Laboratory for Genetic Improvement of Domestic Animal, Hunan Agricultural University, Changsha, China

**Keywords:** Sertoli cells, porcine, proliferation, miR-222, *GRB10*, PI3K/AKT signaling pathway

## Abstract

Sertoli cells are central and essential coordinators of spermatogenesis. Accumulating evidence has demonstrated that miRNAs participate in the regulation of Sertoli cell growth. However, the functions and the regulatory mechanisms of miRNAs in Sertoli cells of domestic animals remain largely unknown. Here we report that miR-222 overexpression repressed cell cycle progression and proliferation and promoted the apoptosis of immature porcine Sertoli cells, whereas miR-222 inhibition resulted in the opposite result. miR-222 directly targeted the 3′-UTR of the *GRB10* gene and inhibited its mRNA abundance. An siRNA-induced *GRB10* knockdown showed similar effects as did miR-222 overexpression on cell proliferation and apoptosis and further attenuated the role of miR-222 inhibition. Furthermore, both miR-222 overexpression and *GRB10* inhibition repressed the phosphorylation of PI3K and AKT, the key elements of the PI3K/AKT signaling pathway, whereas *GRB10* inhibition offsets the effects of the miR-222 knockdown. Overall, we concluded that miR-222 suppresses immature porcine Sertoli cell growth by targeting the *GRB10* gene through inactivation of the PI3K/AKT signaling pathway. This study provides novel insights into the epigenetic regulation of porcine spermatogenesis by determining the fate of Sertoli cells.

## Introduction

Spermatogenesis is an extraordinarily complex and tightly regulated process, which finally produces spermatids with the participation of multiple cell types, including macrophage, endothelial, myoid, Leydig, Sertoli, and innate lymphoid type II cells (Green et al., 2018). Sertoli cells play key roles in guaranteeing normal spermatogenesis by generating a stable microenvironment, maintaining immune tolerance, and secreting several functional proteins for generative cells. Additionally, Sertoli cells are the final targets of follicle-stimulating hormone and testosterone during spermatogenesis (Mancuso et al., 2018). However, each Sertoli cell has a fixed capacity to support the development of germ cells, indicating that Sertoli cell proliferation determines sperm production in adults. Recent studies pointed out that non-coding RNAs [such as microRNA (miRNA)] regulate Sertoli cell proliferation (Papaioannou et al., 2009, 2011; Procopio et al., 2017).

miRNAs are a series of conserved small non-coding RNAs that are widely involved in various physiological processes, including cell proliferation, apoptosis, and differentiation, by targeting the 3′-untranslated region (3′-UTR) of mRNA and thereby causing translational inhibition or activation (Suzuki et al., 2017; Xiao et al., 2017). Selective ablation of Dicer, an RNase III endonuclease required for miRNA biogenesis, in Sertoli cells impeded cell–cell junctions in the seminiferous epithelium (Korhonen et al., 2015), affected the testicular proteome (Papaioannou et al., 2011), and induced progressive testicular degeneration, which further led to male infertility (Papaioannou et al., 2009). Regulatory roles of miRNAs in Sertoli cell proliferation, apoptosis, and synthesis functions were further proposed. For example, miR-202-3p induced Sertoli cell apoptosis and inhibited cell proliferation and synthesis by targeting *LRP6* and *Cyclin D1* of Wnt/β-catenin signaling (Yang et al., 2019). miR-7450 inhibited non-thermal plasma-induced chicken Sertoli cell apoptosis by activating the AMPK signaling pathway (Zhang et al., 2018). miR-301b-3p/3584-5p enhances low-dose mono-n-butyl phthalate-induced Sertoli cell proliferation by targeting *Rasd1* (Yin et al., 2018). However, knowledge of the functions and the regulatory mechanisms of miRNAs in Sertoli cells is still in its infancy, especially regarding porcine Sertoli cell proliferation.

Our previous studies have shown that miR-222, a member of the miR-222 family, exhibits higher expression levels in the neonatal and prepubertal periods of the developing porcine testicular tissues (Ran et al., 2015; Weng et al., 2017b). miR-222 participated in spermatogenesis through maintaining the undifferentiated state of mammalian spermatogonia by repression of *KIT* expression (Yang et al., 2013). In addition, miR-222 could be cloned from purified mice Sertoli cells at P6 (Papaioannou et al., 2009) and function as a regulator of cell proliferation and apoptosis in multiple types of cancer cells (Zeng et al., 2016; Li et al., 2017). These findings suggested that miR-222 might participate in regulating porcine Sertoli cell proliferation; however, the mechanisms involved remain unknown. In the present study, we found that miR-222 inhibited immature porcine Sertoli cell proliferation and promoted apoptosis. miR-222 directly targeted the 3′-UTR of the growth factor receptor-binding protein 10 (*GRB10*) gene and repressed its mRNA abundance. A *GRB10* knockdown offsets the effects of miR-222 inhibition on immature porcine Sertoli cell proliferation. Furthermore, miR-222 inactivated the PI3K/AKT signaling pathway by inhibiting *GRB10* gene expression.

## Materials and Methods

### Cell Culture and Transfection

The commercial swine testis cells (ATCC CRL-1746) isolated from swine 80- to 90-day-old fetal testes have been identified as immature porcine Sertoli cells (Ma C. P. et al., 2016). Additionally, we also detected that the marker genes of Sertoli cells, *SOX9*, *Amh*, and *Wt1*, are specifically expressed in this commercial swine testis cells (Ran et al., 2018). The immature porcine Sertoli cells were cultured in Dulbecco’s modified Eagle medium (HyClone, United States) containing 10% fetal bovine serum (Gibco, Grand Island, United States) at 37°C with 5% CO_2_.

For cell transfection, 100 pmol (final concentration, 50 nM in the cells) miR-222 mimic (GenePharma, China), which mimics a negative control (mimic NC) (GenePharma, China), miR-222 inhibitor (RiboBio, China), inhibitor NC (RiboBio, China), *GRB10* siRNA (RiboBio, China), siRNA NC (RiboBio, China), NC + mimic NC, miR-222 inhibitor + siRNA NC, or miR-222 inhibitor + *GRB10* siRNA was diluted with 250 μl serum-free Opti-MEM (Thermo Fisher Scientific Inc., United States) and incubated at 28°C for 5 min. Then, 5 μl Lipofectamine^TM^ 2000 (Invitrogen, United States) was also diluted with 250 μl serum-free Opti-MEM and incubated at room temperature for 5 min. These two mixtures were mixed and incubated at room temperature for 15 min. Finally, the mixtures were added to each well when the cells reached approximately 80% confluence. After cultivation for 6 to 8 h at 37°C with 5% CO_2_, the complete medium was used for cultivation.

$$f\,\left(x\right)=a_{0}+\sum_{n=1}^{\infty }\left(a_{n}\,\cos\frac{n\,\pi \,x}{L}+b_{n}\,\sin\frac{n\,\pi \,x}{L}\right)$$

### Cell Cycle Assay

The cell cycle was analyzed using a cell cycle testing kit (Nanjing KeyGen Biotech, China) according to the manufacturer’s protocols. After 24 h of transfection, the cells were washed three times with phosphate-buffered saline and harvested in a 1.5-ml centrifuge tube. Then, the cells were incubated in 70% (v/v) ethanol overnight at −20°C and then in propidium iodide (PI) solution (50 mg/ml) for 30 min at 4°C. The cell suspension was analyzed using a FACSCanto II flow cytometer (Becton Dickinson, United States). Three independent replicates were conducted for each cell group.

### CCK-8 Assay

Cells were seeded in a 96-well culture plate at a density of 1 × 10^4^ cells/well in 100 μl of culture medium. For the cell counting kit-8 (CCK-8, Multiscience, China) assay, 10 μl CCK-8 medium was added to each well at 0 (used as the negative control) or 48 h after transfection. Then, the cells were incubated for 4 h at 37°C. The absorbance value of each well was detected using an ELISA plate reader (Molecular Devices, United States) at 450 nm. At least three independent biological replicates were used in this assay.

### EdU Assay

For the 5-ethynyl-2′-deoxyuridine (EdU, RiboBio, China) assay, 100 μl EdU medium (50 μmol) was added to each well 24 h after transfection, and the cells were incubated for 2 h at 37°C. Then, DNA staining solution and EdU staining solution were added to each well to mark living (blue) and proliferating (red) cells, respectively, according to the manufacturer’s protocols. We used a fluorescence microscope to observe the cells at × 20 and ImageJ software (NIH, United States) to determine the cell numbers. At least three independent biological replicates were used in this assay.

### Cell Apoptosis Assay

Cell apoptosis was detected using an Annexin V-FITC apoptosis detection kit (Nanjing KeyGen Biotech, China) and an adenosine triphosphate (ATP) assay kit (Beyotime, China). Cells were cultured in six-well plates with 2 ml of medium. After 24 h of transfection, the cells were collected in a 1.5-ml centrifugation tube. Before Annexin V-FITC apoptosis analysis, the cells were washed three times and double-stained with FITC-Annexin V and PI. The cell samples were analyzed using a FACSCanto II flow cytometer (Becton Dickinson, United States). Percentages of early and late apoptosis cells were counted and used to calculate the cell apoptosis rate. The ATP concentration was evaluated using an ATP assay kit (Beyotime, China), according to the manufacturer’s protocols. The relative ATP levels of the experimental groups were normalized to that of the NC group. Additionally, the protein expression levels of cell survival-related genes (*BCL2*, *BAX*, and *Caspase-3*) were also measured using western blotting.

### Dual-Luciferase Activity Assay

A target site between miR-222 and *GRB10* 3′-UTR was predicted using TargetScan 7.2^1^ and RNAhybrid^2^ online software. The *GRB10* 3′-UTR sequence (wild type or mutant type) was amplified using RT-PCR assay (Supplementary Table S1). Then, they were subcloned into pmirGLO dual-luciferase vectors (Promega, United States). The vectors were co-transfected with miR-222 mimic or mimic NC into immature porcine Sertoli cells. After 48 h of transfection, we measured the luciferase activity of each cell group using the Dual-Glo luciferase assay system (Promega, United States). Renilla luciferase (Promega) activity was used as the internal control.

### Real-Time qPCR

Real-time quantitative PCR (qPCR) was performed as described in our previous studies (Ran et al., 2016, 2017, 2018; Weng et al., 2017a). Total RNA was extracted using TRIzol reagent (Invitrogen, United States), according to the manufacturer’s protocols. The primers were designed using Oligo 7.0 software (Molecular Biology Insights Inc., United States) (Supplementary Table S1) and synthesized by Sango Bio. (Shanghai, China). The cDNA of each sample was synthesized using a PrimeScript first-strand cDNA synthesis kit (TaKaRa, China), according to the manufacturer’s protocols. The qPCR amplifications were performed on a PIKO REAL 96 real-time PCR System (Thermo Scientific, United States) using an SYBR Green kit (TaKaRa, China). All qPCR reactions were performed in triplicate. *U6* and *pig-TBP* genes were used as internal controls for the miR-222 and *GRB10* genes, respectively. The relative expression of each gene was evaluated using the 2^–△△*Ct*^ method.

### Western Blotting

Total cellular protein was extracted using a radioimmunoprecipitation assay lysis buffer (Beyotime, China). The protein concentration was measured using a bicinchoninic acid protein assay kit (Beyotime, China), according to the manufacturer’s protocols. The boiled protein samples were electrophoresed on 10% sodium dodecyl sulfate-polyacrylamide gels and then transferred onto a polyvinylidene fluoride membrane (Beyotime, China). The membrane containing protein fractions was blocked with 5% non-fat milk for 2 h and incubated with primary antibodies overnight at 4°C, including GRB10 (1:500, Merck Millipore, Germany), BCL2 (1:1,000, Proteintech Group, United States), BAX (1:2,000, Proteintech Group, United States), Caspase-3 (1:100, Abcam, Cambridge, MA), p-PI3K (1:1,000, phospho Tyr458, Cell Signaling Technology, United States), p-AKT (1:1,000, phospho Ser473, Affinity, United States), PI3K (1:1,000, Proteintech Group, United States), AKT (1:2,000, Proteintech Group, United States), and β-actin (1:2,000, Proteintech Group, United States). After washing, the membrane was incubated with secondary antibodies (1:5,000, Proteintech Group, United States) for 2 h at room temperature. Protein bands were visualized using an ECL Advanced Western Blotting detection kit (Beyotime, China). β-actin served as the loading control.

### Statistical Analysis

Data are presented as mean ± standard deviation (SD). Data from experiments with multiple cell groups were subjected to a one-way ANOVA, followed by Duncan’s multiple-comparison test of significance using SPSS 17.0 software (IBM, United States). A *t*-test was used to test the differences in the experiment with only two cell groups. *P* < 0.05 or *P* < 0.01 was considered as statistically significant.

## Results

### miR-222 Inhibits Cell Cycle Progression and Proliferation in Immature Porcine Sertoli Cells

**FIGURE 1:**
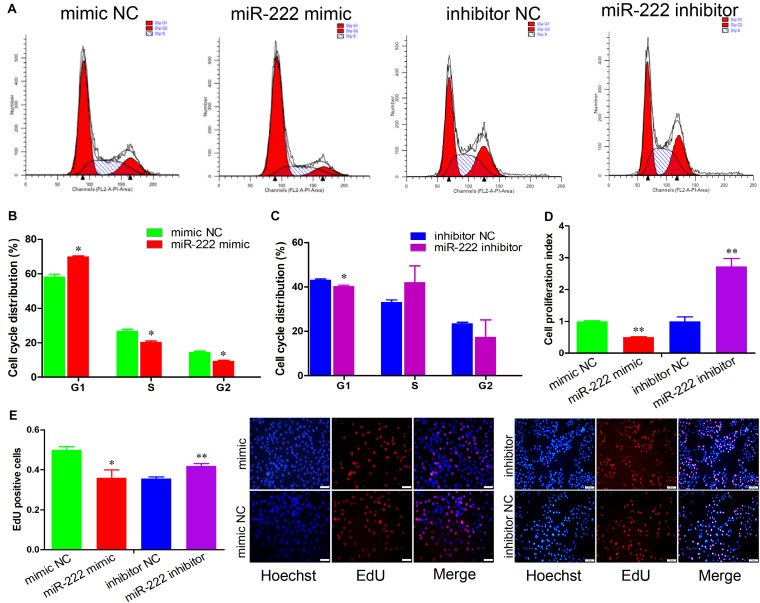
miR-222 represses immature porcine Sertoli cell proliferation. **(A)** The immature porcine Sertoli cells were transfected with mimic NC, miR-222 mimic, inhibitor NC, or miR-222 inhibitor (*n* = 3). The cell cycle was analyzed using a FACSCanto II flow cytometer. **(B,C)** The cell cycle distribution was calculated using the miR-222 mimic **(B)** or miR-222 inhibitor **(C)** transfected cell group (*n* = 3). **(D)** The CCK-8 kit was used to measure the effect of miR-222 on cell proliferation (*n* = 3). The absorbance value of each well was detected using an ELISA plate reader at 450 nm. **(E)** The cell mitotic activity was detected using the EdU corporation assay (*n* = 3). Representative images of EdU staining of immature porcine Sertoli cells 24 h after transfection. Scale bar = 200 μm. Data are presented as mean ± SD. ^∗^*P* < 0.05 and ^∗∗^*P* < 0.01.

To evaluate the effects of miR-222 on immature porcine Sertoli cells, we performed four-cell transfection groups with multiple assays. Transfection efficiency was measured using qRT-PCR, and the results demonstrated that the relative expression of miR-222 was significantly increased by the miR-222 mimic and decreased by the miR-222 inhibitor (*P* < 0.01) (Supplementary Figure S1A). Cell cycle analysis showed that miR-222 overexpression increased the percentage of cells in G1 phase and decreased that in the S and G2 phases compared with that of the mimic NC-transfected cell group (*P* < 0.05) (Figures 1A,B). Conversely, knockdown of miR-222 reduced the G1 phase cell population (*P* < 0.05) (Figures 1A,C). These results indicated that miR-222 arrested cells in the G1 phase and further repressed cell cycle progression.

The role of miR-222 in immature porcine Sertoli cell proliferation was further measured using CCK-8 and EdU incorporation assays. The CCK-8 proliferation assay demonstrated that miR-222 overexpression significantly decreased the cell proliferation index, whereas miR-222 inhibition promoted immature porcine Sertoli cells (*P* < 0.01) (Figure 1D). Similarly, the results from the EdU staining assay also showed that the percentage of EdU-positive cells was significantly lower in the miR-222 mimic-treated cell group than that in the control group (*P* < 0.05), whereas the EdU-positive cell population was significantly higher in the miR-222 inhibitor-transfected cell group than in the inhibitor NC-transfected cell group (*P* < 0.01) (Figure 1E). These results suggested that miR-222 inhibited immature porcine Sertoli cells.

### miR-222 Promotes Immature Porcine Sertoli Cell Apoptosis

**FIGURE 2:**
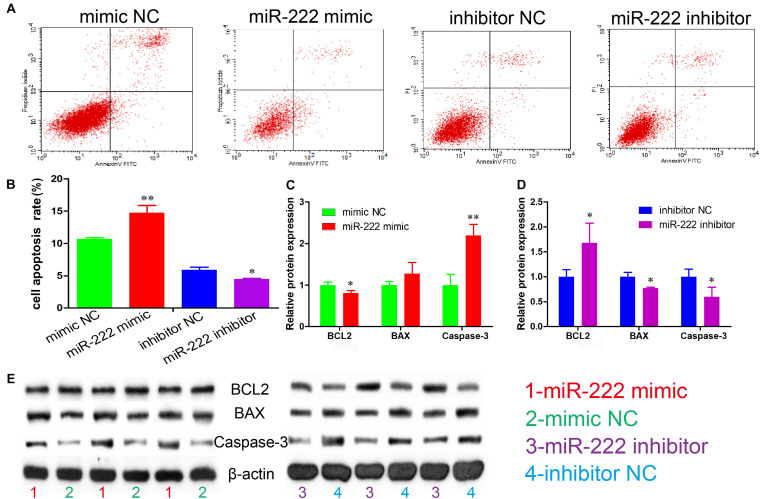
miR-222 promotes apoptosis in immature porcine Sertoli cells. **(A)** The apoptosis phase distributions were identified using the Annexin V-FITC/PI staining assay (*n* = 3). **(B)** The cell apoptosis rate was induced by the miR-222 mimic or the miR-222 inhibitor. **(C,D)** The effect of the miR-222 mimic **(C)** and miR-222 inhibitor **(D)** on the protein expression of cell survival-related genes, BCL2, BAX, and Caspase-3. **(E)** The protein expression of cell survival-related genes was measured using western blot assay. The β-actin gene was used as the internal control. Data are presented as mean ± SD. ^∗^*P* < 0.05 and ^∗∗^*P* < 0.01.

We also examined the regulatory role of miR-222 in cell apoptosis. The results of Annexin V and PI staining and flow cytometry indicated that the cell apoptosis rate was significantly increased in the cell group overexpressing miR-222 (*P* < 0.01) and significantly decreased after miR-222 inhibition (*P* < 0.05) (Figures 2A,B). Furthermore, BCL2 protein expression, an inhibitor of cell apoptosis, was significantly downregulated by miR-222 overexpression (*P* < 0.05), whereas it was significantly upregulated by miR-222 inhibition (*P* < 0.05). miR-222 overexpression increased the protein expression of Caspase-3, an apoptosis indicator (*P* < 0.01), whereas it showed the opposite effects when miR-222 was inhibited in immature porcine Sertoli cells (Figures 2C–E). These results suggested that miR-222 induced immature porcine Sertoli cell apoptosis.

### miR-222 Directly Targets the GRB10 Gene

**FIGURE 3:**
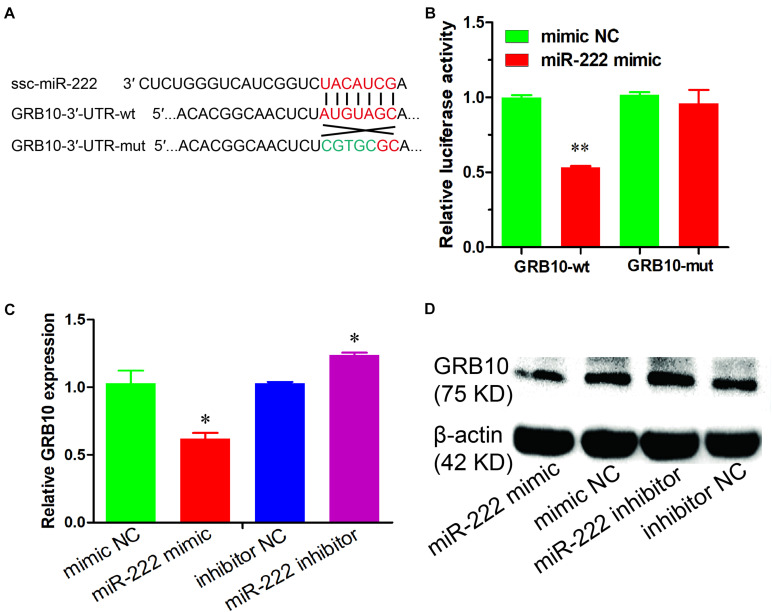
miR-222 directly targets the *GRB10* gene. **(A)** The target site of miR-222 and GRB10 gene was predicted using the TargetScan and RNAhybrid online software. **(B)** Luciferase reporter assay results were measured in immature porcine Sertoli cells with co-transfection of the miR-222 mimic/mimic NC and *GRB10*-3′-UTR-wt/mut vector (*n* = 3). Renilla luciferase (Promega) activity was used as an internal control. **(C,D)** The *GRB10* mRNA and protein expressions were detected using qRT-PCR **(C)** and western blot **(D)** assays, respectively. *pig-TBP* and β-actin were used as internal controls, respectively. Data are presented as mean ± SD. ^∗^*P* < 0.05 and ^∗∗^*P* < 0.01.

To investigate the potential molecular mechanism underlying the effects of miR-222 on the proliferation and the apoptosis of immature porcine Sertoli cells, the *GRB10* gene was predicted as a potential target gene of miR-222 using TargetScan online software. We constructed the 3′-UTR of the *GRB10* dual-luciferase reporter vector with the wild type (wt) and mutant type (mut) and then co-transfected them into immature porcine Sertoli cells with either the miR-222 mimic or the mimic NC. The results indicated that miR-222 overexpression significantly suppressed the relative luciferase activity of the *GRB10*-wt reporter vector (*P* < 0.01) but had no effect on the *GRB10*-mut reporter vector. However, mimic NC did not influence the luciferase activity of both of the abovementioned reporter vectors (Figure 3A). Additionally, the mRNA and protein expression of the *GRB10* gene was significantly downregulated by miR-222 overexpression and upregulated in response to miR-222 inhibition (Figures 3B,C). These results showed that miR-222 directly targeted the *GRB10* gene and further repressed its mRNA abundance.

### GRB10 Knockdown Inhibits Immature Porcine Sertoli Cell Proliferation and Induces Apoptosis

**FIGURE 4:**
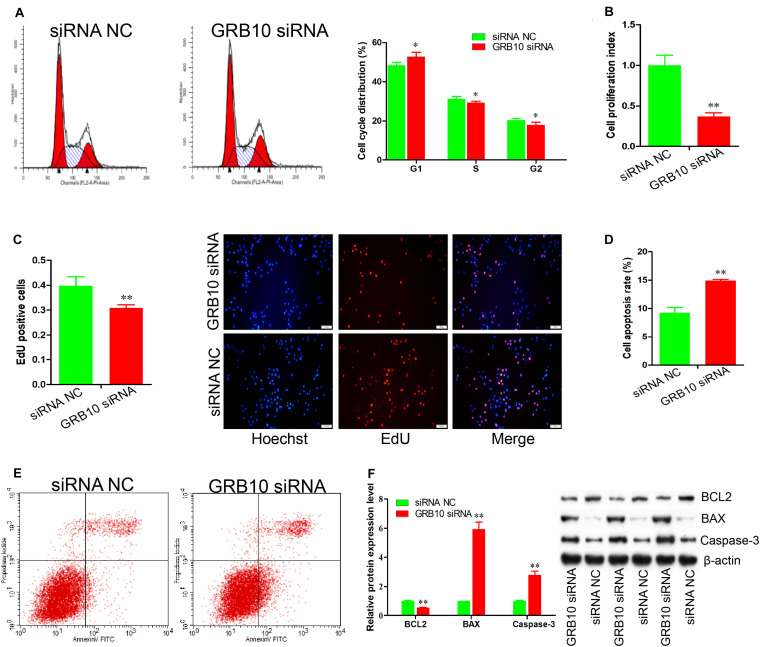
*GRB10* deficiency inhibits proliferation and induces apoptosis in immature porcine Sertoli cells. **(A)** The cell cycle was analyzed using a FACSCanto II flow cytometer, and its distribution was calculated (*n* = 3). **(B,C)** The effects of *GRB10* inhibition on cell proliferation was detected using CCK-8 **(B)** and EdU corporation **(C)** assays (*n* = 3). Scale bar = 100 μm. **(D,E)** The cell apoptosis rate was measured using the Annexin V-FITC/PI staining assay (*n* = 3). **(F)** The protein expression of cell survival-related genes was measured using western blot assay. The β-actin gene was used as the internal control. Data are presented as mean ± S.D. ^∗^*P* < 0.05 and ^∗∗^*P* < 0.01.

To explore the regulatory roles of the *GRB10* gene in immature porcine Sertoli cells, a specific siRNA was transfected to knock down the expression of the *GRB10* gene (*P* < 0.01) (Supplementary Figure S1B). The cell cycle analysis results revealed that the *GRB10* knockdown resulted in an increase in the G1 phase cell population but a decline in the cell population in the S and G2 phases (*P* < 0.05) (Figure 4A), which indicated that *GRB10* inhibition repressed cell cycle progression by arresting cells in the G1 phase. To determine cell proliferation, we used the CCK-8 assay and found that cell proliferation was inhibited by the siRNA-induced *GRB10* inhibition (*P* < 0.01) (Figure 4B). The EdU incorporation assay also indicated that knockdown of the *GRB10* gene significantly decreased cell mitotic activity (*P* < 0.01) (Figure 4C).

We further established the effect of *GRB10* on cell apoptosis. The Annexin V and PI staining assay demonstrated that the apoptosis rate in the cell group with GRB10 siRNA was significantly higher than that in the siRNA NC-transfected cell group (*P* < 0.01) (Figures 4D,E). Furthermore, the *GRB10* knockdown significantly decreased BCL2 protein expression (*P* < 0.01) and significantly increased the protein expression of BAX and Caspase-3 (*P* < 0.01) (Figure 4F). These results showed that *GRB10* knockdown inhibited immature porcine Sertoli cell proliferation and induced cell apoptosis.

### GRB10 Knockdown Attenuates the Effects of miR-222 Inhibition on Immature Porcine Sertoli Cells

**FIGURE 5:**
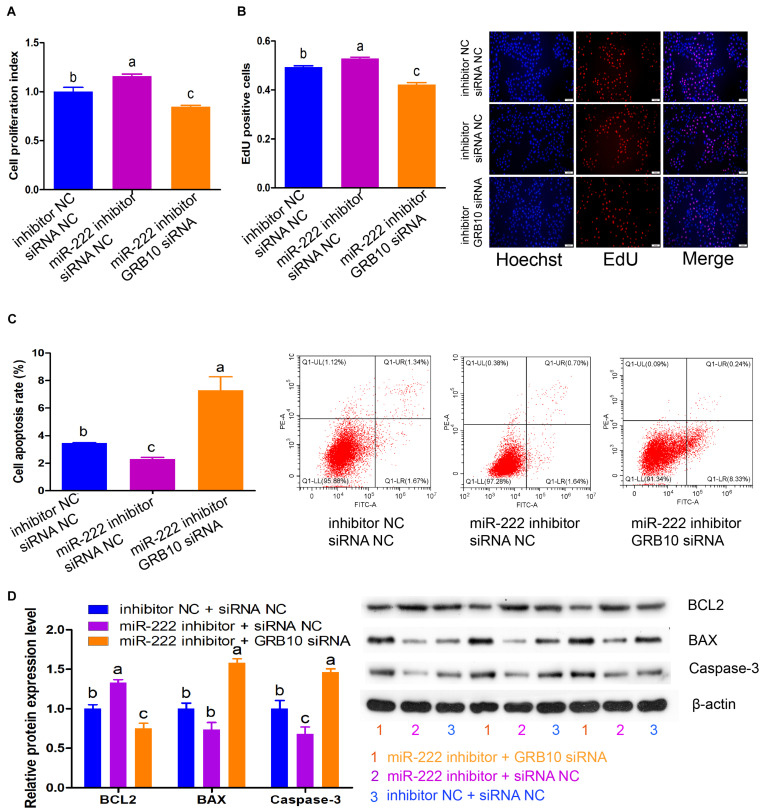
*GRB10* knockdown attenuated the effects of miR-222 inhibition. Three co-transfection treatments were constructed in this experiment, including inhibitor NC + siRNA NC, miR-222 inhibitor + siRNA NC, and miR-222 inhibitor + *GRB10* siRNA. **(A,B)** The effects of these three co-transfection treatments on cell proliferation were detected using CCK-8 **(A)** and EdU corporation **(B)** assays (*n* = 3). Scale bar = 200 μm. **(C)** The cell apoptosis rate was measured using the Annexin V-FITC/PI staining assay (*n* = 3). **(D)** The protein expression of cell survival-related genes was measured using western blot assay. The β-actin gene was used as the internal control. Data are presented as mean ± SD. ^∗^*P* < 0.05 and ^∗∗^*P* < 0.01.

These results demonstrated that *GRB10* inhibition induced similar effects as miR-222 overexpression on cell proliferation and apoptosis. Therefore, we constructed three co-transfection treatments to validate that the *GRB10* gene mediated the regulatory roles of miR-222, including the inhibitor NC + siRNA NC, miR-222 inhibitor + siRNA NC, and miR-222 inhibitor + *GRB10* siRNA. The CCK-8 and EdU incorporation assays showed that cell proliferation activity was significantly increased by the miR-222 inhibitor + siRNA NC treatment (*P* < 0.05), whereas it was reduced by the miR-222 inhibitor + *GRB10* siRNA treatment compared with that of the NC + siRNA NC treatment (*P* < 0.05) (Figures 5A,B). Additionally, detection during the Annexin V-FITC/PI staining assay showed that miR-222 inhibition induced a lower cell apoptosis rate, which was significantly increased after translation with *GRB10* siRNA (*P* < 0.01) (Figure 5C). Similarly, miR-222 inhibition significantly increased BCL2 protein expression and decreased the protein expression of BAX and Caspase-3 (*P* < 0.05), whereas these effects were offset by the *GRB10* knockdown (*P* < 0.05) (Figure 5D). Taken together, *GRB10* knockdown antagonized the effects of miR-222 inhibition in immature porcine Sertoli cells.

### miR-222 Inactivates the PI3K/AKT Signaling Pathway by Downregulating GRB10 Expression

**FIGURE 6:**
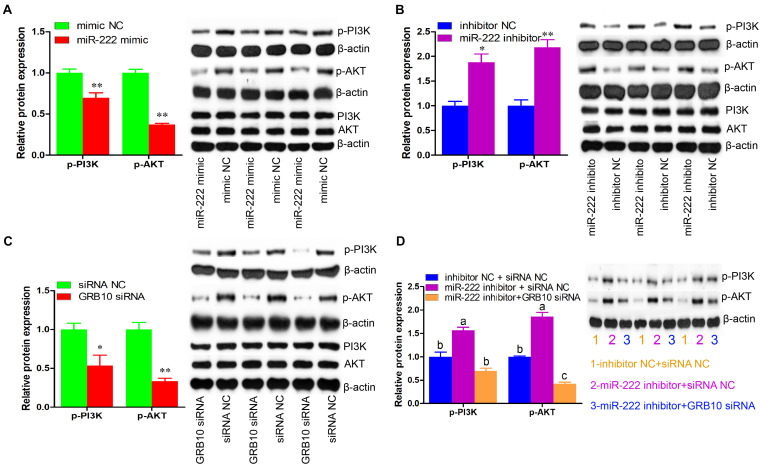
miR-222 inactivates PI3K/AKT signaling pathway through the downregulation of *GRB10* expression. The p-PI3K (phospho Tyr458), p-AKT (phospho Ser473), PI3K, and AKT protein levels were detected using western blot assay (*n* = 3). The β-actin gene was used as the internal control. **(A–C)** The effects of miR-222 mimic **(A)**, miR-222 inhibitor **(B)**, and *GRB10* siRNA **(C)** on the p-PI3K and p-AKT protein levels. **(D)** The *GRB10* knockdown attenuated the effects of miR-222 inhibition on the p-PI3K and p-AKT protein levels. Data are presented as mean ± SD. ^∗^*P* < 0.05 and ^∗∗^*P* < 0.01.

We then measured the effects of miR-222 on the phosphorylation of PI3K and AKT, the key elements of the PI3K/AKT signaling pathway. Compared with the controls, the expression of p-PI3K and p-AKT was significantly decreased by miR-222 overexpression (*P* < 0.01) (Figure 6A), whereas their expression was elevated by miR-222 inhibition (*P* < 0.05) (Figure 6B). siRNA-induced *GRB10* inhibition significantly reduced the expression of p-PI3K and p-AKT, which was similar to that of miR-222 overexpression (*P* < 0.05) (Figure 6C). Additionally, *GRB10* inhibition attenuated the effects of miR-222 inhibition on the expression of p-PI3K and p-AKT (Figure 6D). Collectively, these data demonstrated that miR-222 inactivated the PI3K/AKT signaling pathway by downregulation of *GRB10* expression.

## Discussion

Sertoli cells are the central and essential coordinators of spermatogenesis. In pigs, the intense proliferation of Sertoli cells in the prepubertal period determines their final number in adults, which further affects sperm production and male fertility. Recently, several miRNAs have been implicated in the regulation of porcine Sertoli cell proliferation and apoptosis *via* different target genes and signaling pathways, including miR-638 (Hu et al., 2017), miR-762 (Ma C. et al., 2016), miR-196a (Zhang S. et al., 2019), miR-1285 (Jiao et al., 2015), miR-499 (Gao et al., 2019), miR-26a (Ran et al., 2018), and miR-34c (Ran et al., 2019). However, hundreds of mature miRNAs have been detected in developing porcine testicular tissues. Therefore, further studies are needed to explore the functional roles of miRNAs in porcine Sertoli cells (Luo et al., 2015, 2018; Li et al., 2016; Xu et al., 2018). In the present study, we report that miR-222 inhibited cell cycle and proliferation and induced cell apoptosis in immature Sertoli cells by targeting the *GRB10* gene through the inactivation of the PI3K/AKT signaling pathway.

miR-222 has been reported as a key regulator of the proliferation of multiple cell types. For instance, miR-222 inhibits the proliferation of ovarian cancer cells (Fu et al., 2016) and nucleus pulposus cells (Wang W. et al., 2019), whereas it promotes proliferation in gastric cancer cells (Li et al., 2017), pulmonary arterial smooth muscle cells (Xu et al., 2017), prostate cancer cells (Wang et al., 2015), and primary mouse hepatocyte cells (Higashi et al., 2019). In this study, the results from CCK-8 and EdU incorporation assays indicated that overexpression of miR-222 decreased cell proliferation, and miR-222 inhibition resulted in the opposite effect. These findings demonstrated that the effect of miR-222 on cell proliferation was cell type dependent. Additionally, miR-222 affects the cell cycle by regulating the expression of the cell cycle inhibitor p27(kip1) in human thyroid papillary carcinomas (Visone et al., 2007), mast cells (Mayoral et al., 2009), and human hepatocellular carcinoma cells (Dai et al., 2010). We determined that miR-222 arrested cells in the G1 phase and further repressed cell cycle progression. These findings indicated that miR-222 repressed immature porcine Sertoli cell proliferation partly by impeding cell cycle progression. Furthermore, we also detected the effects of miR-222 on immature Sertoli cell apoptosis. In the BCL2 family, BCL2 (an anti-apoptotic protein) and BAX (the pro-apoptotic protein) have opposite effects on regulating the mitochondria to release the caspase activators (Volkmann et al., 2014). The caspase protease family (such as Caspase-3) will be activated when the BAX-to-BCL2 ratio is increased. It then catalyzes the specific cleavage of many key cellular proteins, leading to apoptosis (Horbay and Bilyy, 2016). In this study, we observed that miR-222 overexpression resulted in a decrease in BCL2 expression and an increase in protein expression of Caspase-3, which further contributed to the cell apoptosis rate, whereas miR-222 inhibition showed the opposite effect. Taken together, miR-222 inhibited the cell cycle and proliferation and promoted apoptosis in immature porcine Sertoli cells.

Hundreds of the target genes of miR-222 were predicted using the TargetScan online software, and the *GRB10* aroused our attention as it is involved in the regulation of gene transcription and translation, the cell cycle, and cell growth and proliferation by inducing the PI3K-AKT signaling pathway (Kazi and Ronnstrand, 2013; Khan et al., 2019; Zhang T. et al., 2019). Furthermore, it has been reported that LY294002-induced inhibition of the PI3K/AKT signaling pathway inhibits porcine Sertoli cell proliferation and promotes cell apoptosis (Gao et al., 2019). Therefore, the *GRB10* gene was set as a potential target of miR-222 to explore the mechanism of miR-222 in the regulation of proliferation and apoptosis in immature porcine Sertoli cells. In our study, the *GRB10* gene was further confirmed as a target of miR-222 using bioinformatic analysis and dual-luciferase assay. Furthermore, miR-222 repressed mRNA abundance for the *GRB10* gene. The *GRB10* gene, a member of the GRB7 family of adaptor molecules, has been shown to participate in the regulation of cell proliferation and apoptosis by associating with multiple proteins and signaling molecules (Han et al., 2001). For example, GRB10 increases DNA synthesis through the upregulation of growth factors, PDGF BB, IGF-I, and insulin (Kabir and Kazi, 2014). GRB10 also increases cell survival by interacting with the anti-apoptotic mitochondrial Raf targeted by Bcl-2 and the putative phosphorylation sites on the dynein binding domain of Bim (Hu et al., 2010). Additionally, miR-199a inhibits cell proliferation and promotes cell apoptosis by targeting *GRB10* (Xia et al., 2014). In the present study, the siRNA-induced *GRB10* knockdown inhibited cell proliferation and promoted cell apoptosis in immature porcine Sertoli cells and further abolished the effects of miR-222 inhibition.

Previous studies have shown that GRB10 functions as a regulator of the PI3K/AKT signaling pathway through the regulation of PI3-kinase catalytic activity and AKT phosphorylation (Jahn et al., 2002; Deng et al., 2003; Khan et al., 2019). In this study, we found that miR-222 overexpression or *GRB10* inhibition induced a decrease in the phosphorylation levels of PI3K and AKT, whereas the miR-222 knockdown showed the opposite effect. Furthermore, *GRB10* inhibition offsets the regulatory role of the miR-222 knockdown on the phosphorylation of PI3K and AKT. Recently, it has been widely reported that the PI3K/AKT signaling pathway is involved in the proliferation and apoptosis of Sertoli cells. Thyroid hormone, follicle-stimulating hormone, and dibutyl phthalate inhibit cell proliferation or induce cell apoptosis in Sertoli cells through the repression of the PI3K/AKT signaling pathway (Riera et al., 2012; Sun et al., 2015; Wang C. et al., 2019), whereas 17β-estradiol promotes Sertoli cells by activating this pathway (Yang et al., 2017). Additionally, the PI3K/AKT signaling pathway also mediates the effects of miR-638 and miR-499 on the proliferation and apoptosis of immature porcine Sertoli cells (Hu et al., 2017; Gao et al., 2019). Based on these abovementioned clues and our results, we concluded that miR-222 inhibited proliferation and induced apoptosis in immature porcine Sertoli cells through the inactivation of the PI3K/AKT signaling pathway.

## Conclusion

Collectively, this study provided evidence that miR-222 suppressed immature porcine Sertoli cell growth by targeting the *GRB10* gene through the inactivation of the PI3K/AKT signaling pathway. We suspected that miR-222, *GRB10*, and the PI3K/AKT signaling pathway were involved in porcine spermatogenesis *via* the control of Sertoli cell number and sperm production.

## Data Availability Statement

The raw data supporting the conclusions of this article will be made available by the authors, without undue reservation.

## Author Contributions

MR and BC contributed to the experimental conception and design. FP, HL, MR, BW, XT, YC, and AY performed the experiments. FP and HL analyzed the data and wrote the first draft of the manuscript. MR revised the manuscript. All the authors have reviewed and approved the final manuscript.

## Conflict of Interest

The authors declare that the research was conducted in the absence of any commercial or financial relationships that could be construed as a potential conflict of interest.

## Footnotes

1 http://www.targetscan.org/vert_72/

2 https://bibiserv.cebitec.uni-bielefeld.de/rnahybrid/

## Supplementary Material

Supplementary Figure 1 The transfection efficiency of miR-222 and GRB10. **(A)** The immature porcine Sertoli cells were transfected with mimic NC, miR-222 mimic, inhibitor NC, and miR-222 inhibitor (*n* = 3). The relative expression of miR-222 was measured using a qRT-PCR assay. *U6* was used as the internal control. **(B)** siRNA NC and GRB10 siRNA were, respectively, transfected in the immature porcine Sertoli cells (*n* = 3). The relative mRNA expression of the *GRB10* gene was detected using the qRT-PCR assay. *pig-TBP* was used as an internal control. Data are presented as mean ± SD. ^∗^*P* < 0.05 and ^∗∗^*P* < 0.01.

Supplementary Table 1 The sequences of the primers used in this study.
