# Supplementary figures and images for: miR-222 Suppresses Immature Porcine Sertoli Cell Growth by Targeting the GRB10 Gene Through Inactivating the PI3K/AKT Signaling Pathway
Source: Front Genet. 2020 Oct 29;11:581593. doi: 10.3389/fgene.2020.581593 (PMC7673446; doi:10.3389/fgene.2020.581593)

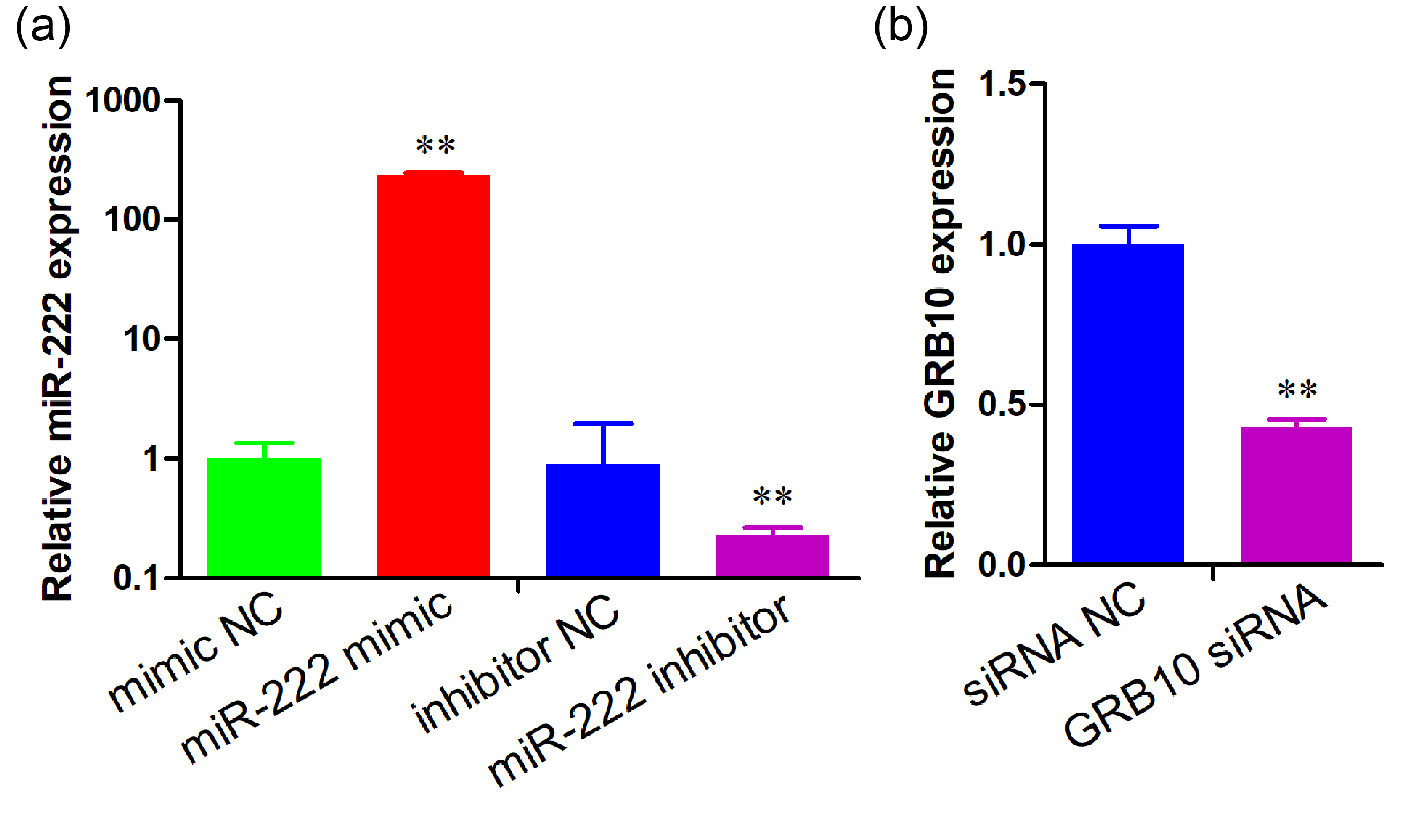

Supplement: Supplementary Figure 1 — The transfection efficiency of miR-222 and GRB10. (A) The immature porcine Sertoli cells were transfected with mimic NC, miR-222 mimic, inhibitor NC, and miR-222 inhibitor (n = 3). The relative expression of miR-222 was measured using a qRT-PCR assay. U6 was used as the internal control. (B) siRNA NC and GRB10 siRNA were, respectively, transfected in the immature porcine Sertoli cells (n = 3). The relative mRNA expression of the GRB10 gene was detected using the qRT-PCR assay. pig-TBP was used as an internal control. Data are presented as mean ± SD. ∗P < 0.05 and ∗∗P < 0.01. [file Image_1.TIF]
