# Supplementary material for: miR-222 Suppresses Immature Porcine Sertoli Cell Growth by Targeting the GRB10 Gene Through Inactivating the PI3K/AKT Signaling Pathway
Source: Front Genet. 2020 Oct 29;11:581593. doi: 10.3389/fgene.2020.581593 (PMC7673446; doi:10.3389/fgene.2020.581593)
Supplement: Supplementary Table 1 — The sequences of the primers used in this study. [file Table_1.DOCX]

Table S1 Synthetic oligo sequences

| Gene | Primer | Sequence (5’→3’) |
| --- | --- | --- |
| *Pig-TBP* | F-Primer | GCGATTTGCTGCTGTAATCA |
|  | R-Primer | CCCCACCATGTTCTGAATCT |
| *GRB10* | F-Primer | TTGCACCATCCGTACTACCA |
|  | R-Primer | ACGTCATCCTCTTGGTGAGG |
| U6 | RT-Primer | AACGCTTCACGAATTTGCGT |
|  | F-Primer | CTCGCTTCGGCAGCACA |
|  | R-Primer | AACGCTTCACGAATTTGCGT |
| ssc-miR-222 | RT-Primer | GTCGTATCCAGTGCAGGGTCCGAGGTATTCGCACTGGATACGACGAGACC |
|  | F-Primer | ATCGGAGCTACATCTGGCTACTG |
|  | R-Primer | AACGCTTCACGAATTTGCGT |
| *GRB10*-wt | F-Primer | GCTGAAGGACATGCGACTTT |
|  | R-Primer | CTGCATTCCCTGAAAACGAT |
| *GRB10*-mut | F-Primer | TGCGCACGAGAGTTGCCGTGT |
|  | R-Primer | CGTGCGCAAAACCCTCCCCTG |
